# The evolutionary origins of the Global Neuronal Workspace in vertebrates

**DOI:** 10.1093/nc/niad020

**Published:** 2023-09-13

**Authors:** Oryan Zacks, Eva Jablonka

**Affiliations:** The Cohn Institute for the History and Philosophy of Science and Ideas, Tel Aviv University, Ramat Aviv 6934525, Israel; The Cohn Institute for the History and Philosophy of Science and Ideas, Tel Aviv University, Ramat Aviv 6934525, Israel; CPNSS, London School of Economics, Houghton St., London WC2A 2AE, United Kingdom

**Keywords:** minimal consciousness, minimal GNW, Unlimited Associative Learning, animal consciousness, event memory, hippocampus, fish neuroanatomy

## Abstract

The Global Neuronal Workspace theory of consciousness offers an explicit functional architecture that relates consciousness to cognitive abilities such as perception, attention, memory, and evaluation. We show that the functional architecture of the Global Neuronal Workspace, which is based mainly on human studies, corresponds to the cognitive-affective architecture proposed by the Unlimited Associative Learning theory that describes minimal consciousness. However, we suggest that when applied to basal vertebrates, both models require important modifications to accommodate what has been learned about the evolution of the vertebrate brain. Most importantly, comparative studies suggest that in basal vertebrates, the Global Neuronal Workspace is instantiated by the event memory system found in the hippocampal homolog. This proposal has testable predictions and implications for understanding hippocampal and cortical functions, the evolutionary relations between memory and consciousness, and the evolution of unified perception.

## Introduction: the Global Neuronal Workspace and Unlimited Associative Learning theories of consciousness

There are currently several theories trying to explain different facets of consciousness and synthesize them under a unified framework (for recent reviews, see Doerig et al. 2021; Seth and Bayne 2022; Yaron et al. 2022). The majority of these theories are concerned with human consciousness and suggest that a “core network,” the location and properties of which are hotly debated, instantiates consciousness (Koch et al. 2016; Melloni et al. 2021). These theories have overlapping and complementary assumptions, and although all recognize that evolutionary analysis can shed light on the study of consciousness, the evolutionary perspective has received little attention. In this section, we focus on one of the leading theories of consciousness today, the Global Neuronal Workspace (GNW) theory (Mashour et al. 2020), which puts forward a functional architecture relating consciousness to key cognitive abilities (perception, attention, motor control, memory, and evaluation). We argue that the GNW architecture aligns with the Unlimited Associative Learning (UAL) model (Ginsburg and Jablonka 2019), suggesting a path for the evolution of consciousness in vertebrates. We then reconstruct the GNW architecture in basal vertebrates (section “The neural functional architecture of the first jawed vertebrates”) and show that the event memory system of basal vertebrates has a dual role—acting both as a memory system and as a global workspace (section “The dual role of the hippocampal homolog: both a memory system and a Global Neuronal Workspace”). We end with a discussion of the functional and evolutionary implications and predictions stemming from this proposal (section “Discussion”).

### The Global Neuronal Workspace theory of consciousness

The GNW theory is based on the global workspace theory put forward by Baars (1988), who emphasized the distinction between two modes of information processing: conscious and unconscious. Unconscious processing is carried out by multiple modular and parallel processors in the brain, each devoted to a specific set of computations and localized in a restricted neural network. Conscious processing is the result of integration and broadcast by the global workspace, a cognitive architecture that has limited informational capacity, so that conscious experience is singular, unified, sequential, and carries only a small number of items at any given moment (Baars 2005).

Dehaene et al. (1998) suggested a “neuronal” implementation of the global workspace: for an object to be perceived consciously, it must be “globally available to multiple brain systems through a network of neurons with long-range axons densely distributed in prefrontal, parieto-temporal, and cingulate cortices” (Mashour et al. 2020, p. 776). This network enables the integration of information from different sources into a unified representation that can be sustained over time. To provide an evolutionary analysis of the GNW theory, we focus on four main principles that are consistent throughout the development of the theory and need to be addressed by any evolutionary account of the GNW.

#### A network structure with central hubs involved in multimodal sensory integration

The GNW theory proposes a neural architecture with many subnetworks involved in different computations. Within these subnetworks, pyramidal neurons with long-range connections have high connectivity to neighboring local cells, as well as pyramidal cells found in other, distant subnetworks. The long-range pyramidal neurons create a horizontal brain-scale network so that they receive information from other processors and, when appropriate, broadcast information from their local subnetwork across the brain to other subnetworks. These pyramidal neurons provide integration and coordination between different functional processors. Most of these neurons are inhibited most of the time, but the small subset that is active determines the contents of consciousness.

According to this proposal, the global workspace is not located in a specific brain area but is distributed across the neocortex and perhaps other key structures. However, areas with especially high numbers of long-range pyramidal neurons are considered central hubs (hence the emphasis on prefrontal, cingulate, and parietal cortices). These regions participate in a larger percentage of global activity and perform computations that may regulate, or be necessary, for many instances of global broadcast (Dehaene and Changeux 2011). Network hubs are critical for fast communication, integration, and synchronization across different processors, and facilitate gating to the global workspace. Once the network is stabilized around these hubs, losing them will cause disproportionate damage to the overall functioning of the network (van den Heuvel and Sporns 2013; Mashour et al. 2020).

#### Five main categories of local processors

**Figure 1.**
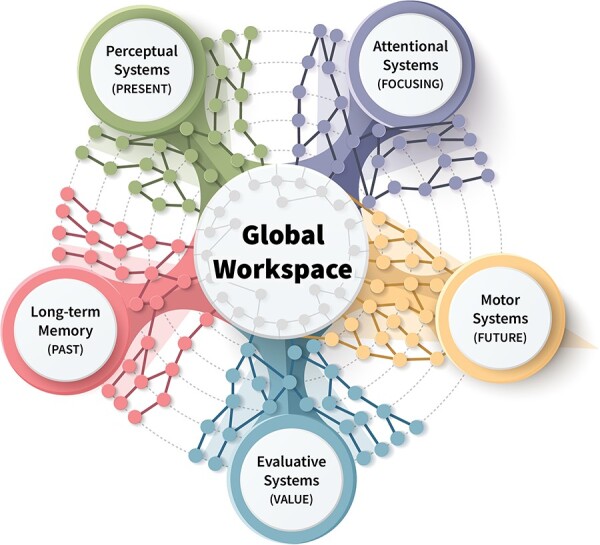
The GNW model: The major categories of parallel processors are connected to the global workspace; local processors have specialized operations, but when they access the global workspace, they share information, hold it, and disseminate it (figure is based on Dehaene et al. (1998))

Dehaene et al. (1998) divided the many possible local processors into five categories: perception, motor control, memory, value, and attention (Fig. 1). These processors can be dynamically mobilized into the global workspace, contributing to conscious content. Dehaene et al. (1998) have not discussed explicitly why these five categories were chosen and if they are meant to be exhaustive and non-redundant. They also did not comment on the extent of integration within each category, before the information reaches the global workspace. They have, however, provided general localizations for the different categories in humans and implicitly in other mammals (e.g. perceptual circuits are attributed to occipital, temporal, and parietal cortical areas). The local processors they identified are also those pointed to by learning theorists. To account for learning, interactions among sensory, motor, memory, and reinforcement processors are necessary, and attention processes are essential.

#### Distinctive activation dynamics from subprocessors to Global Neuronal Workspace neurons and between these neurons

Local processors gain access to the global workspace by the non-linear process of ignition. When a processor performs unconscious computations, its activity remains local and fades over short time periods. For the contents of the processor to be broadcast globally, its information is propagated in a feed-forward manner, resulting in fast, coherent, and exclusive activation of a subset of GNW neurons, while all others are inhibited. The information can be sustained, thanks to recurrent activity among GNW neurons and local processors. “Active workspace neurons send top-down amplification signals that boost the currently active processor neurons, whose bottom-up signals in turn help maintain workspace activity” (Dehaene and Naccache 2001, p. 19).

#### The sequential nature of conscious experiencing is shaped by neuronal bottleneck dynamics

Any aspect of our environment that enters our awareness is immediately integrated into the ongoing experience. However, the number of details we can represent at any given moment is extremely limited in comparison to the endless inputs available in the environment and in comparison to the amount of detail processed unconsciously in our brain. In his book on the subject, Dehaene (2014) proposed that the neural architecture giving rise to consciousness imposes constraints on experiencing, generating a bottleneck through which only a limited number of details can be consciously represented. If consciousness is instantiated by a coherent brain-scale network (the GNW), many connections in this network must undergo inhibition to sharpen the signal and distinguish it from noise. Inhibition and amplification at the local level determine the contents that may gain access to the global workspace. Similarly, global inhibition and amplification between GNW neurons enable the maintenance of a unified representation over time, as well as shifts from one conscious experience to the next. Additionally, this limited arena may represent only the most salient information at a relevant level of abstraction and granularity to enable effective decision-making.

When considering the GNW in humans and other mammals, it may seem obvious that all local processors, as well as the GNW itself, are organized across the neocortex (Dehaene et al. 2017). However, from most current evolutionary perspectives, minimal consciousness vastly predates the evolution of mammals endowed with a neocortex (Merker 2007; Ginsburg and Jablonka 2007a, b; Barron and Klein 2016; Feinberg and Mallatt 2016). If this is indeed the case and if the GNW theory is a general framework for consciousness, how can consciousness become implemented in animals that lack a neocortex, such as birds, reptiles, or fish? How did the evolution of the neocortex transform consciousness in mammals? If the GNW theory suggests a general architecture that instantiates consciousness in all conscious animals, not just mammals, we expect to find neural structures and dynamics that are functionally similar to the GNW in animal groups that lack a neocortex. Such structures were suggested by the learning-based theory advocated by Ginsburg and Jablonka (2019), which focuses on minimal consciousness.

### The Unlimited Associative Learning theory of consciousness

Ginsburg and Jablonka (2019) compiled a list of characteristics that are jointly sufficient for minimal consciousness and can be described in neural, cognitive, behavioral, and phenomenological terms. Their list was meant to reflect the current consensus regarding the neurological bases of consciousness, incorporating elements of the global workspace theory, as well as other theories (see Ginsburg and Jablonka 2019, Chapter 3). As they described it, minimal consciousness is first-order consciousness that does not require metacognition and is characterized by the following capacities: unification and differentiation, global accessibility and broadcast, temporal depth, flexible value attribution, attention processes, mapping capacity, goal-directed voluntary behavior, and self–other distinction from a point of view. Based on this list of characteristics, Ginsburg and Jablonka identified an evolutionary transition marker for minimal consciousness. This marker has a diagnostic capacity that requires that all the consensus properties that are jointly sufficient to attribute minimal consciousness to an entity are in place, thus indicating that the transition to consciousness has gone to completion (this methodology is based on Gánti’s approach to the origins of life; Gánti 2003).

Unlimited associative learning (UAL), a domain-general, open-ended form of associative learning, was suggested as the transition marker of minimal consciousness. This type of learning, which includes both world- and self-learning, is operationalized by the following, testable learning capacities: (i) discrimination learning—learning to discriminate among differently organized, novel, multi-featured patterns of sensory stimuli and between novel, composite action patterns; (ii) trace conditioning—the capacity to reliably learn about a predictive neutral stimulus or an action pattern even when there is a time gap between the presentation of the predictive stimulus or action and its reinforcement; (iii) learn to flexibly alter the evaluation of predictive stimuli and action patterns, which enables the animal to make motivational trade-offs, prioritizing different outcomes in a context-sensitive manner; and (iv) second-order conditioning—learning about the predictive value of new stimuli or action on the basis of previously learned stimuli and actions. Ways of testing the hypothesis that all four UAL capacities jointly entail minimal consciousness can be found in Birch et al. (2020).

**Figure 2.**
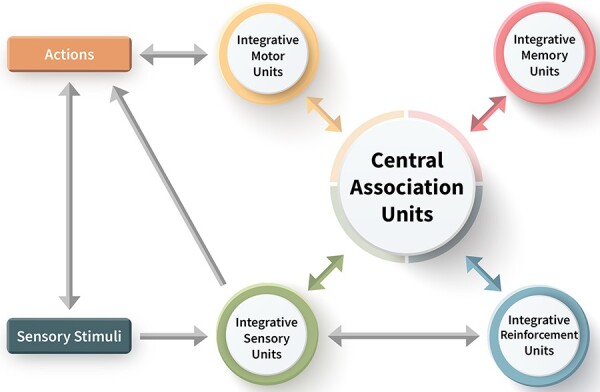
A minimal toy model of the UAL architecture: UAL is hypothesized to depend on reciprocal connections between sensory, motor, reinforcement (value), and memory processing units, which come together to construct a central association unit, depicted at the core of the network (figure is based on Ginsburg and Jablonka (2019)).

The UAL theory incorporates a functional architecture that closely corresponds to the GNW architecture (Fig. 2). It includes sensory processing units, a dedicated memory subsystem that stores event representations (a precursor of episodic memory; Tulving 2002), a dedicated evaluation subsystem that can assign valence to any compound input configuration and that enables context-sensitive prioritization, and a motor subsystem based on body mapping allowing the representation of prospective actions. All these subsystems must come together within a common neural space, the central association unit. Selective stabilization, involving amplifications and inhibitions that underlie attentional processes, and re-entrant connections and predictive coding are critical to the dynamics of this system.

According to the UAL model, all the attentional functions that are necessary for consciousness (e.g. selective attention, internal attention, and vigilance) are implemented by local processes at different scales, primarily amplification and inhibition between neuronal networks involving negative and positive feedback interactions. This selectivity, which implements an attentional function, does not require a dedicated and specialized attention network (something like a theater spotlight system). Gating (selecting only a small subset of incoming inputs to be globally broadcasted, while all others are suppressed) is achieved both with widespread inhibition applied to the specialized workspace neurons and via inhibitory feedback from these neurons back to the local processors. Therefore, according to the UAL model, attentional processes are a consequence of the local dynamics of the global workspace, with excitation of one network leading to the inhibition of a competing network, based on relative salience. The GNW and UAL theories thus diverge regarding the need for dedicated attentional networks. While such networks might be necessary for human consciousness, a functional model of minimal consciousness can be constructed without them. Table 1 compares the central assumptions of the GNW and UAL theories.

**Table 1.** Similarities and differences between the GNW and UAL theories

Assumptions about structures and functions	GNW theory	UAL theory
	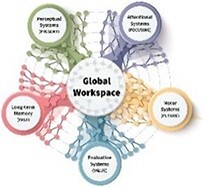	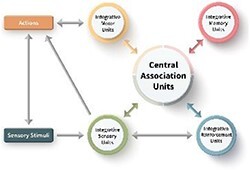
Specific neuronal types are involved in consciousness	Layer II/III excitatory pyramidal neurons with long-range projections	Not committed to a specific neuronal type
Local processors contributing to conscious contents	Perception, motor control, memory, value, and attention	Perception, motor control, memory, and value
Shared principles of brain organization and neural dynamics	Hierarchical organization, recurrent dynamics, and predictive processing	Hierarchical organization, recurrent dynamics, and predictive processing
Brain structures play a special role in consciousness	Prefrontal, anterior cingulate, and parietal cortices	Minimal consciousness can be realized in a plurality of brain structures
The role of attention in conscious processing	Dedicated attention networks are necessary	Dedicated attention networks are unnecessary; inhibition and amplification dynamics within the different networks are sufficient
The role of learning in conscious processing	The theory does not comment on learning processes, but global broadcasting facilitates a wide range of cognitive functions	The architecture supporting consciousness was selected for enabling UAL
Animal groups endowed with conscious experience	The theory is not committed to a specific phylogenetic distribution; primates are considered conscious, and there is limited speculation regarding other mammals and birds	Vertebrates, coleoid cephalopods, and some arthropods

Ginsburg and Jablonka (2019) suggested that the evolutionary emergence of consciousness was driven by the evolution of UAL, which enables animals to flexibly adapt to their environment and make better decisions based on past experiences. Limited associative learning, from which UAL evolved, is an ancient and widely distributed characteristic of many animal phyla. It does not entail consciousness since it does not require (among other things) complex discrimination learning, working memory, and representations of the relations between action outcome and outcome rewards. UAL, on the other hand, does entail minimal consciousness, as it operationalizes all the capacities of minimal consciousness we listed earlier. Since the UAL model is based on learning models, which specify the direction and the temporal order among sensory, motor, memory, and value processors as a core feature of conscious systems, it complements the GNW theory. Moreover, the UAL model suggests a behavioral measure that serves as a criterion for determining the extent of integration within and between the subsystems that leads to minimal consciousness: integration must be sufficient for the animal to exhibit UAL. Importantly, neither theory suggests that the neocortically distributed GNW or the central association unit in the UAL model is the “seat of consciousness.” According to both theories, consciousness is a system property, requiring the dynamic interactions of hierarchical re-entrant connections between sensory, value, memory, and motor units.

## The neural functional architecture of the first jawed vertebrates

**Figure 3.**
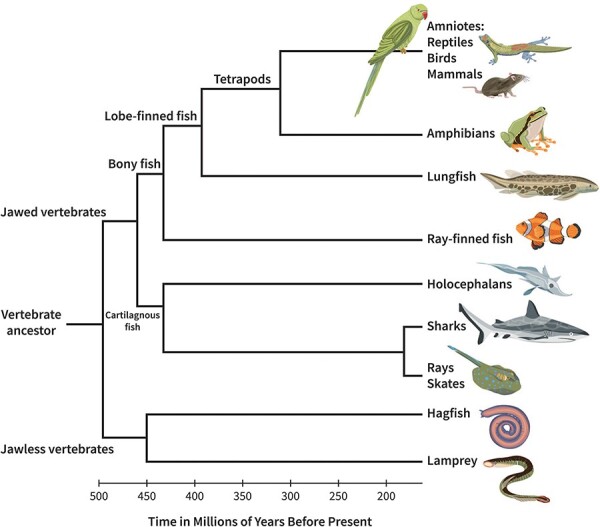
The phylogenetic tree of vertebrates. A major landmark of vertebrate evolution was the development of jaws. Today, only two jawless vertebrate lineages remain: the hagfish and the lampreys. During the Ordovician era, jawed vertebrates are believed to have diverged into three major lineages. First, cartilaginous fish split off, giving rise to modern-day sharks and rays. Subsequently, bony fish diverged into ray-finned fish and lobed-finned fish. Ray-finned fish are a large and diverse group, containing ∼99% of all known fish species. Nearly 400 million years ago (during the Devonian era), a species of lobed-finned fish left their aquatic environment and gave rise to all land vertebrates (tetrapods), which include amphibians, reptiles, birds, and mammals.

To account for the origins of minimal consciousness in all vertebrates, both the GNW and the UAL models need to assimilate what has been learned about the evolution of the vertebrate brain. Based on our current understanding of brain evolution in vertebrates, we suggest a minimal GNW/UAL architecture that can be implemented in a hypothetical basal vertebrate, with a brain thought to reflect the ancestral condition of all major fish groups (Fig. 3; Striedter and Northcutt 2020). Most information on brain organization in basal vertebrates is based on the comparative neuroanatomy of jawed fish, and therefore, this paper focuses on this species-rich taxon rather than the two extant groups of highly specialized jawless vertebrates (Suzuki 2021).

**Figure 4.**
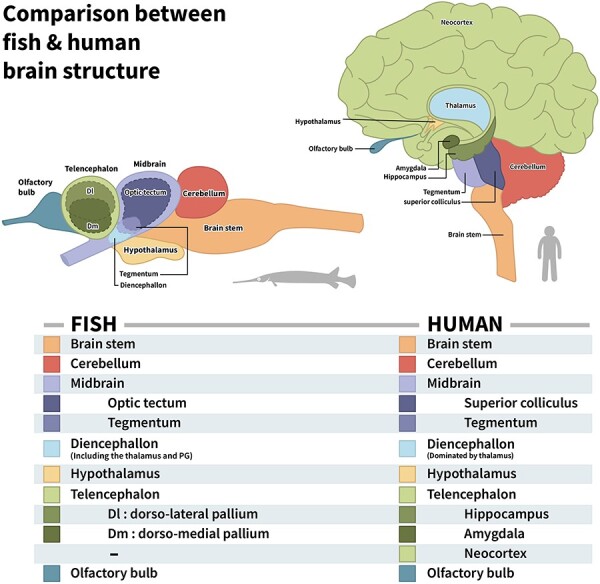
A schematic comparison between fish and human brain structure. Homologous structures are highlighted with similar colors. The neocortex dominates the human brain, but its homology to telencephalic structures in fish (the covering around the dorsolateral and dorsomedial pallium) is still debated. The diencephalon is situated between the midbrain and the telencephalon and mediates the connections between them. PG, preglomerular complex. The fish brain is based on illustrations of a longnose gar brain (Striedter and Northcutt 2020)

It is only by aggregating information from many basal lineages together that we can identify the structures of early fish brains. For the ray-finned fish, the most basal group is the Polypteriformes; for the cartilaginous fish, it would be fruitful to study holocephalans such as ratfish although more information about brain and behavior is needed for both sharks and rays. Coelacanths and lungfish are the only remaining fishes in the lobbed-finned lineage. A study of their brains, as well as those of amphibians, is necessary if we are to gain more insight into the ancestors of tetrapods. The fish brain shown in Fig. 4 is inspired by the brain of a longnose gar (a basal lineage of ray-finned fish; Striedter and Northcutt 2020, p. 131) and is meant to represent a general Bauplan of jawed fish brains.

While lacking a neocortex, the study of extant and fossil fish suggests that vertebrate ancestors had neural networks dedicated to sensory perception, motor control, value, and event memory (Fig. 4; Broglio et al. 2005; Vernier 2016; Briscoe and Ragsdale 2019). If these processors interact to form an integrated architecture consistent with that suggested by the GNW/UAL theories, it is critical then to analyze their functions, their neural instantiations, and their relationship with a global workspace. We therefore start with a short discussion of these subsystems in basal jawed fish.

### Sensory subsystems

In basal jawed vertebrates, the midbrain tectum is the primary destination of visual information from the retina, combined with other sensory modalities such as auditory, somatosensory, electrosensing, and lateral line sensing (Striedter and Northcutt 2020). There are indications that the non-visual modalities align spatially with visual information so that the tectum processes information according to its location in space. This structure is highly developed in several lineages, especially teleost fish, which may have up to 15 distinct layers in their tectum. This network has direct connections to motor areas in the tegmentum, medulla, and spinal cord. Striedter and Northcutt (2020) suggest that a primary function of the tectum is coordinating eye and head movements, directing orientation toward or away from salient stimuli in the animal’s immediate environment. Tectal lesions severely impair the animal’s ability to orient, in some cases even more so than removing the eyes, reflecting its role in integrating multimodal sensory information. The tectum is connected to the telencephalon via the diencephalon providing integrated sensory input, primarily to the hippocampal homolog (HH). The major component of the diencephalon in amniotes is the thalamus, while in ray-finned fish the preglomerular complex is prominent. In both cases, incoming sensory information is processed in these centers before reaching telencephalic structures. In all fish, the HH integrates multimodal sensory information, and many species developed additional telencephalic areas that specialize in sensory processing and feed into the HH.

In all vertebrates, there are two, rather than one, sensory integration areas, a well-known fact that is overlooked by most theories of consciousness including the GNW and the UAL theories (although see Feinberg and Mallatt 2016, Chapter 6). In basal fish, the optic tectum receives and integrates virtually all modalities of sensory information, except olfaction. The olfactory bulb of vertebrates is located in the anterior (frontal) tip of the brain and projects directly to the pallium of the telencephalon. It is thought that in early vertebrates, most of the telencephalon was dedicated to processing olfactory information (Striedter and Northcutt 2021). The telencephalic pallium and the midbrain tectum are separated by the diencephalon, which mediates most of their communication. The telencephalon processes olfactory information and uses it to affect the animal’s behavior so that any such information reaching the tectum is already processed.

The early vertebrate’s pallial telencephalon did not receive direct, unprocessed, non-olfactory sensory information (Northcutt et al. 2004). In early vertebrates, as in extant jawed fish, there were two rather than one major integrating, sensory processors, olfaction and all other sensory modalities. Even in mammals, olfactory information does not pass through the thalamus before reaching the cortex, in contrast to all other senses. This points to the possibility of decoupling conscious olfactory experience from tectal processing so that integrated olfactory information in an intact animal may become conscious independently of the integration of information from other sensory modalities. This may be especially evident in basal vertebrates, which lack processing of non-olfactory sensory information in additional pallial territories before reaching the HH. However, the extent of decoupling critically depends on the extent of integration in the HH, so this remains an open question.

### Motor control subsystem

The motor system is the final integrator of any information directly affecting behavior. In both the GNW and UAL models, it is visualized as the single and major output unit of the cognitive architecture in a conscious animal. However, the motor system is also an important source of input to the global workspace since it broadcasts efferent motor plans and receives feedback from other processors over many iterations (Keller and Mrsic-flogel 2018). In the GNW theory, this idea is expressed through the centrality of recurrent processing in the overall neural dynamics of consciousness. The UAL model highlights the crucial role of motor representation for reafference, and the learning based on it, with an animal being able to account for its own movements, distinguish their effects from those independent of its actions, and remember them when they lead to a rewarding outcome.

Primary motor areas project to spinal motor neurons that directly innervate body muscles. Secondary, or higher, motor processors in the brain project to primary motor areas but do not have direct connections to the spinal cord. This basic structure is conserved across all vertebrates although lineages differ in the exact locations of primary and secondary areas (Feinberg and Mallatt 2016). In basal vertebrates, the primary motor areas are grouped at the base of the brain and primarily include the medulla and nuclei located in the midbrain tegmentum (Nudo and Frost 2009; Vernier 2016). Secondary motor areas include the cerebellum (which developed in jawed vertebrates), midbrain tectum, hypothalamus, and limited areas of the diencephalon and telencephalic subpallium (the basal ganglia; Moreno et al. 2017; Yopak et al. 2020). These areas modulate motor output through their projections to primary motor areas. Their computations incorporate incoming sensory information, and they use efferent motor copies to differentiate between expected changes in sensory input (due to the animals’ movements) and unexpected input that carries new information about the environment. Recurrent activity is possible through the feedback connections from spinal motor neurons to primary motor areas and from primary and secondary motor areas to other brain processors (Barkan and Zornik 2019).

### Value subsystem/s

The value system serves for representing homeostasis, monitoring any departure from it, and guiding decision-making (Panksepp 2011; Carvalho and Damasio 2021). This system is “hard-wired” in the sense that no learning is needed for certain states or stimuli to be inherently valued as good or bad. However, this system is incredibly flexible since it allows for many new things to be learned on the inherited scaffold of innate values, and under some conditions, innate predispositions can be suppressed. Finally, there is extensive integration within this system so that all the animals’ motivations and needs are accounted for and prioritized, with the most important need fulfilled first, while other action plans are suppressed.

Several theories of affect suggest that the origin of the value system is in interoception, with specialized neurons sensing and monitoring internal states of the body (temperature, pressure, and chemical levels; Denton et al. 2009; Carvalho and Damasio 2021; Solms 2021). When a departure from homeostasis is detected, the animal must take action to return to a homeostatic attractor state. One very important area for such functions is the brainstem, which includes the substantia nigra, raphe nucleus, ventral tegmental area, and periaqueductal gray. Some of these nuclei have sensory specializations (for instance, periaqueductal gray receives nociceptor information and mediates pain) and others secrete unique neuromodulators to wide areas of the brain (ventral tegmental area and substantia nigra release dopamine, and the raphe nucleus releases serotonin). These systems are found in basal fish and are conserved across all vertebrate lineages (Vernier 2016; Pérez-Fernández et al. 2017; Martínez-García and Lanuza 2018).

Another important interoceptive center is the hypothalamus, which is conserved across all vertebrates (Moreno et al. 2017; Striedter and Northcutt 2020). The hypothalamus is known for its connections with the endocrine system, directly sampling the bloodstream and secreting a variety of molecules that relate to and regulate physiological states such as hunger, thirst, and temperature, as well as sexual behavior. Other important centers of the value system are found in the telencephalon, especially in the subpallium. The subpallial basal ganglia (such as the striatum) are considered homologous across all vertebrates and are a major target of brainstem neuromodulators, such as dopamine and serotonin (Redgrave et al. 2010; Lanciego et al. 2012). Finally, the amygdala is also considered part of the value system, and it contains both subpallial and pallial divisions (Pessoa et al. 2019). Cortical value centers, such as the orbitofrontal cortex, developed along with the rest of the neocortex only in mammals.

In basal vertebrates, the amygdala processes mostly olfactory information, with other types of sensory information (visual, auditory, and tactile) being processed and evaluated in the midbrain (Medina et al. 2017). To integrate crucial olfactory information and modulate behavior according to it, basal vertebrates had some level of value representation close to the primary olfactory sensory areas. It seems that an ancient feature of amygdala neurons is their receptiveness to sexual hormones, pointing to some of the key features of interoceptive neurons (Lanuza et al. 2008). Both the subpallial amygdala and striatum project to important centers in the hypothalamus and brainstem, including the midbrain tegmentum, modulating motor behavior.

### Event memory subsystem

In their original paper, Dehaene et al. (1998) described the declarative memory system as providing access to past percepts and events. Here, we focus on perceptual event memories that require the percept or the action is remembered in context. The context need not include a temporal, narrative dimension (i.e. it does not require the “when” aspect in the what-where-when dimensions; Tulving 2002). It is recognized that a specialized subnetwork in the vertebrate telencephalon, the mammalian hippocampus and its non-mammalian homologs (HH), is dedicated to the formation and storage of such event memories (Allen and Fortin 2013). Such memories can be learned after a single exposure, or very few trials, and can contain spatial or contextual aspects of the event (see Zacks et al. (2022) for a recent review of animal experimental data on such memory abilities).

The mammalian hippocampus is a unique and specialized structure, but many of its features can be identified in anamniote homologs. For example, in weakly electric fish, electrosensory information passes several layers of processing (including in the midbrain and diencephalon) before reaching the HH (Trinh et al. 2016). Like the dentate gyrus (part of the mammalian hippocampus), the main field of sensory input into the fish HH contains the largest number of neurons out of all layers of the processing hierarchy (Giassi et al. 2012). This enables a sparse and independent representation of different stimulus features, which can become associated when they co-occur. Reminiscent of the mammalian CA3 hippocampal field, fish HH has substantial recurrent connectivity within its network, which enables encoding, storage, and retrieval. Finally, the HH has feed-forward connections that may make extensive use of synaptic modulation for the final storage of learned associations. In some fish, HH projects to a smaller network (dorso-central) that serves as a contractive funnel and projects outside the pallium, eventually reaching motor areas and affecting behavior. It is important to note that in mammals, the hippocampus interacts with the neocortex and many other structures to support diverse and complex forms of memory. Since basal fish lack a neocortical homolog, they probably have a different division of labor between the components of the memory system, leading to heavier reliance on the HH for memory storage.

### Global information processing in basal vertebrates

**Figure 5.**
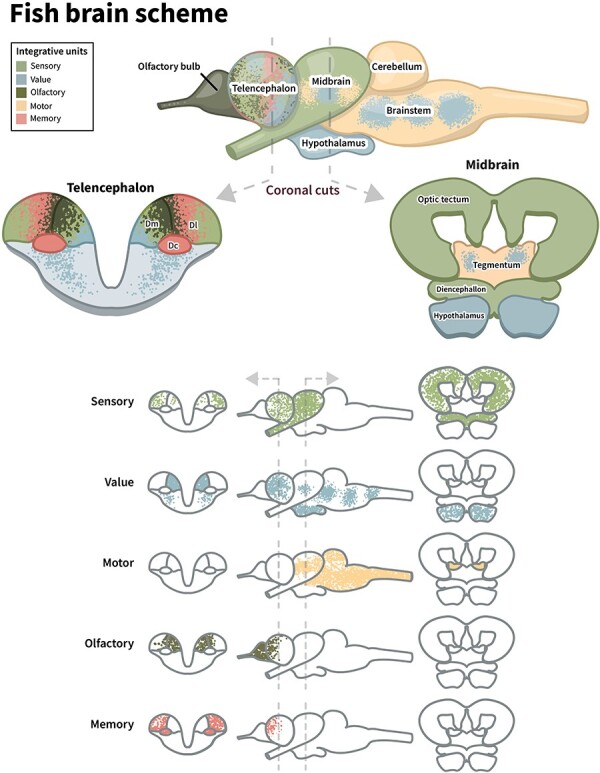
A schematic summary of GNW components in the brain of a basal fish. The figure highlights the structures most involved in the different functional networks. The figure is based on illustrations of a longnose gar brain (Striedter and Northcutt 2020)

The four major components (sensory, motor, value, and memory) identified in basal vertebrates correspond to the mammalian subsystems in the GNW and UAL models. However, as we noted, two sensory integrating processors rather than one must be considered, with the telencephalon processing olfactory information and the midbrain tectum processing information from all other senses. The value system seems relatively diffused, with important centers found near the two major sensory processors (in the midbrain and telencephalon). The motor subsystem can be clearly identified in the brainstem, and the most important network for event memory in fish is located in the HH (part of the telencephalon). These subsystems are described in Fig. 5. But where is the global workspace or the integrating associative hub in these fish? Does one of the subnetworks have a dual function, acting not only as a dedicated processor but also as a GNW? We suggest that the HH is a major hub of the minimal vertebrate GNW.

## The dual role of the hippocampal homolog: both a memory system and a Global Neuronal Workspace

The anatomical structure most closely resembling a global workspace in basal vertebrates is the HH. Considering the evolution of the telencephalon, the HH was the first section that lost strong olfactory projections and gained more diverse input from other pallial and subpallial areas. It serves as the highest level of integration in the fish brain and is the first to develop complex local intra-connectivity and longer efferent projections outside of the telencephalic pallium, primarily to the subpallium and hypothalamus (Striedter and Northcutt 2020). We therefore suggest that in ancestral vertebrates (and extant anamniotes), the long-term memory processor and the global workspace overlapped. In line with this proposal, Howard Eichenbaum concluded 40 years of research on the hippocampus thus: “It is not too simplistic to conclude that the hippocampal network reflects all the salient events in attended experience, just as it should as indicated by its core function in memory” (Eichenbaum 2017, p. 87). We suggest that only later during phylogeny, as information processing capacities progressively evolved in mammals (as well as birds and perhaps some later fish lineages), dedicated structures developed, and the memory and workspace functions became anatomically and functionally separated. The hippocampus, which was previously a central hub of GNW, became specialized for fast and flexible memory encoding, while the neocortex provided additional layers of integration and higher-order representation.

In agreement with this proposal, a combination of nascent neocortical and hippocampal features has been found in the HH. The anterior commissure links the two hemispheres of the fish telencephalon and includes direct connections between the HH of each hemisphere (Fenlon et al. 2021). This is somewhat analogous to mammalian connectivity, in which the corpus callosum connects the neocortical portions of the hemispheres, and the hippocampal commissure provides a direct communication route between the two hippocampi. Two types of connectivity patterns can be identified in the fish HH: an internally recurrent network that could enable pattern separation and completion and neurons with larger, descending axons that use synaptic plasticity to store or strengthen memories (Trinh et al. 2019). Additionally, at any given moment, only a small subset of neurons is active, due to high spiking thresholds, while the rest are inhibited. Trinh et al. (2016) found that the HH of fish has both cryptic layers and columns, along with recurrent and feed-forward connectivity, and that these features resemble the “sensory cortex, [prefrontal cortex] and hippocampus” (Trinh et al. 2016, p. 426).

It would be ideal to examine HH features of the basal species of each major vertebrate lineage. However, in many cases, we were unable to find detailed neuroanatomical descriptions or lesion studies in these animals. We highlight some of the major diagnostic features of the GNW that are manifest in the HH of several well-studied ray-finned fishes.

### Diagnostic features of a global neuronal workspace

#### A network with central hubs connected to all major categories of local processors

In fish, sensory areas (visual, auditory, and lateral line) project to the HH, where they are integrated with olfactory information and input from other networks (such as the hypothalamus and amygdala value systems). The HH is also a major source of pallial output that eventually reaches motor areas to affect behavior. The broadcast aspect of the GNW is reflected in bidirectional interactions with the sensory, value, and motor areas. The HH therefore seems like the best candidate for a central hub of a brain-scale network that integrates information from many other subnetworks, broadcasts it, and has a disproportionate impact on global states.

While central hubs play a crucial role within the GNW network, specialized processors are key contributors of content to global broadcast. Although virtually all vertebrate lineages developed sensory areas in their telencephalic pallium (Suryanarayana et al. 2020; Striedter and Northcutt 2021), we cannot limit ourselves to considering only pallial areas as participating in the global workspace; structures such as the midbrain and diencephalon must be included. Although the HH is the central hub, the GNW is actually more distributed. Nevertheless, the evidence reviewed so far does point to the majority of global workspace information being represented by the GNW neurons located in the telencephalic pallium. Hence, when attempting to decode the contents of conscious experience, pallial neurons (primarily in the HH) are expected to be the best targets of investigation.

#### Distinctive activation dynamics from subprocessors to Global Neuronal Workspace neurons and within Global Neuronal Workspace hubs

The GNW theory predicts fast feed-forward propagation from lower- to higher-level layers and slower recurrent and feedback connections that maintain activity and process it in multiple iterations constraining lower levels through top-down control. The fish HH implements these properties due to its recurrent internal connectivity and widespread pallial connectivity (Elliott et al. 2017). Significant synaptic plasticity utilizing N-methyl-D-aspartate (NMDA) glutamate receptors has been found in fish HH, a hallmark of neocortical association areas and the mammalian hippocampus (Harvey-Girard et al. 2007). NMDA receptors are thought to play an important role in global workspace activity (Mashour et al. 2020).

The GNW theory also posits ignition dynamics, a non-linear process that leads to rapid, coherent, and exclusive activation of a subset of GNW neurons while inhibiting the rest. Proponents of the theory discuss ignition exclusively in the neocortex, but it can be attributed to hippocampal activity, both during memory encoding and retrieval (Moutard et al. 2015; Staresina and Wimber 2019). Specifically, the process of pattern completion in the hippocampal CA3 network is characterized by rapid activation of an attractor pattern, while all other neurons are inhibited. To our knowledge, ignition dynamics have not been investigated in anamniotes. However, similarities in the underlying neural connectivity in the relevant areas of fish and mammals lead to the prediction that such dynamics are likely to be found, especially during tasks that require complex learning and decision-making (Rodríguez et al. 2021).

#### A bottleneck, limiting the amount of information represented at any given moment

The HH is a good candidate for the implementation of an informational bottleneck, thanks to its particular input–output connectivity. In anamniotes, the HH receives inputs from many different brain structures and is considered the main output source of the pallium (in many ray-finned fish, this is mediated by subarea dorso-central, Dc). Thus, most information processed in the pallium that will directly impact behavior must pass through the fish HH (Northcutt 2011). Recurrent connections within the network make it more cohesive so that HH neurons can be considered as a singular representational “set” with limited content capacity.

### An updated model of a minimal Global Neuronal Workspace in basal vertebrates

**Figure 6.**
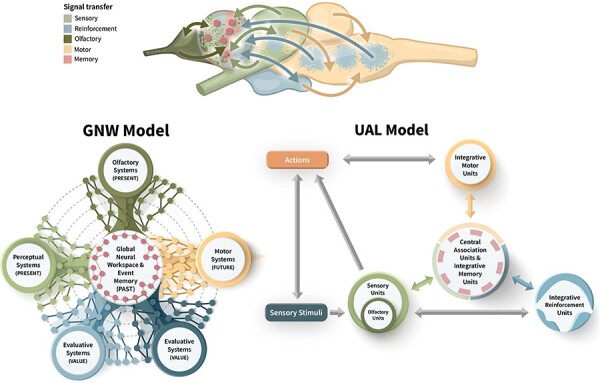
The minimal GNW and UAL systems in the fish brain. Following the analysis of the functional architecture in basal fish brains (top; only some of the re-entrant connections between processors are shown), the figure shows our proposed amendments to the GNW and UAL models for minimal consciousness. In the GNW model, (left) attention functions are instantiated by the internal dynamics of each network and do not have a separate, dedicated subprocessor. The olfactory system is separate from the other sensory modalities, and there is more than one integrating value system (two such systems are shown). The global workspace and event memory system are one and the same. In the UAL model (right), olfaction is separated from the other sensory modalities, and there are several value systems that interact with the integrating units. The central association unit and the integrative memory unit are one and the same

We suggested that the GNW in mammals can be traced back in evolution to the HH in early vertebrates and that it received integrated sensory inputs from two independent sensory processors and diffuse value processors. Although these suggestions do not challenge the basic assumption of the GNW theory and are in line with the UAL model, both the original GNW and UAL models need to be amended to reflect the early stages in the evolution of vertebrate memory and consciousness (as shown in Fig. 6).

As we noted in the first section, the functional architecture of both models is very similar, suggesting an intimate relation between minimal consciousness as conceptualized by the GNW theory and the capacity for open-ended learning, as conceptualized by the UAL model. We suggest that the relationship is evolutionary and functional: the event memory system represents multimodal valued and prioritized information and is therefore central both for the capacity for UAL and for the implementation of a GNW. Given the structural–functional similarity and the tremendous selective advantages of UAL, the suggestion that it drove the evolution of a minimal GNW is, we believe, compelling.

## Discussion

One of the strengths of the GNW theory is the intimate relationship it posits between consciousness and cognition, which offers a basis for many testable predictions. The UAL theory, developed by interrogating the ancient evolutionary origins of consciousness, also claims that a certain learning (cognitive-affective) architecture entails consciousness, and it too provides abundant testable predictions. Our suggested amendments to both GNW and UAL models have implications for our understanding of current and past hippocampal and cortical functions, the conceptualizations of memory and consciousness, and the evolutionary relationships between them. They also point to more testable predictions and open new avenues of research.

### Relations between memory and minimal consciousness

In their summary of a detailed analysis of the fish pallium, Elliott et al. (2017) write: “DD/DL/DC circuitry resembles that of both hippocampal and cortical circuitries. We propose that this distinction may be artificial and that, in teleosts, the processing functions of cortex via its laminar and columnar connectivity and the mnemonic functions of hippocampus may be combined. If this is correct, then DL might be homologous, in whole or in part, to both cortex and dentate gyrus” (p. 43). This supports our proposal that in ancestral and many extant fish, a common neural network has served as both an event memory processor and a global workspace. The separation between integration and mnemonic functions may be artificial in the sense that it is not necessary to support minimal consciousness.

Several other theories recognize the entanglement of learning for conscious processes. Dickinson and Balleine (2010) suggested that goal-directed learning, requiring a representation of the contingency between action and outcome, and outcome as a goal for the agent (anchored in the existential needs of the animal), entails a phenomenal conscious experience (see Jablonka and Ginsburg (2023) for a discussion). Cleeremans (2011) suggested a “higher-order” theory of consciousness, regarding reflection as necessary for conscious experiences. More recently, he and his colleagues proposed that the brain learns to redescribe its own activity to itself, transforming unconscious first-order representations into conscious meta-representations (Cleeremans et al. 2020). Although we accept that learning-based processes, which require hierarchical organization of neural representations, are central to the generation of consciousness during evolution, the requirement for second-order, reflection-requiring representations does not tally with what we have learned about the evolution of consciousness and its taxonomic distribution.

Budson et al. (2022) propose that consciousness is the remembering of first-order representations of unconscious percepts, decisions, and actions and that the function of consciousness is to enable episodic recall. However, episodic noetic and autonoetic memory, which involves metacognition and the experiencing of a remembered or imagined event, requires the addition of new layers and specialized subprocessors to the processing hierarchy underlying minimal consciousness (Zacks et al. 2022). This adds robustness to the network since there can be storage of redundant copies and, crucially, enables enhanced flexibility and nuance so that the impact of a certain memory on the animals’ behavior can be modulated in many different contexts. However, we think that minimal consciousness preceded the evolution of full-blown episodic memory, and the neural mechanisms supporting UAL and minimal consciousness were necessary precursors of episodic memory and imaginative consciousness.

### Major transitions in memory abilities and conscious experience

Our focus in this paper has been minimal consciousness, mainly phenomenal experiences that are strongly anchored to ongoing perceptual information. However, as argued by Ginsburg and Jablonka (2019), this is only the first level of consciousness. Some minimally conscious lineages evolved over time imaginative consciousness and, finally, in the *Homo* genus, symbolic consciousness. The evolution of a planning-enabling, episodic memory system in mammals and birds marks, in our view, a major evolutionary transition, which required the separation of the previously unified memory and consciousness functions of the hippocampus (Zacks et al. 2022). Separating the hippocampal memory system from the neocortical global workspace enabled faster memory formation with enhanced flexibility and processing capacity. This is because the memory system can process information in parallel with the other subnetworks of the architecture, and not every representation within the network requires full ignition and brain-wide broadcast. Perhaps more importantly, an independent memory system could gain access to the global workspace, giving rise to a distinct phenomenal experience of “remembering.” This would enable workspace contents to be internally generated and detached, even for very short intervals, from sensory input. Such independence marks a significant shift from minimal, sensory-based conscious experience to more complex, independent virtual experiences.

Some theories of consciousness consider the thalamocortical system to be necessary and central for conscious processes (Tononi and Edelman 1998; Baars et al. 2013). Thalamocortical connections are thought to modulate the state of consciousness and provide a major communication route between different cortical areas (Aru et al. 2019). This system is found only in mammals and probably developed as the dorsal pallium expanded and gave rise to the neocortex. Although in all vertebrates the thalamus (and other diencephalic structures) project to the pallium, these projections carry mostly sensory information from the midbrain; they do not connect between different pallial areas, and they are not as elaborate as in mammals (Striedter and Northcutt 2020). Thus, like Merker (2007), we think that minimal consciousness can arise without the thalamocortical system, but it may be necessary for higher levels of consciousness. Furthermore, olfactory information reaches the pallium or cortex directly in all vertebrates, without passing through the thalamus. This may indicate that even in mammals, thalamocortical connections are not needed for some types of experiencing, namely olfactory experiencing.

As we noted, the mammalian hippocampus is intimately connected with the thalamus and other diencephalic structures (such as the mammillary bodies; Vertes 2015; Bubb et al. 2017). Some of these connections are clearly evolutionarily conserved since even in vertebrates lacking a neocortical homolog (anamniotes), the HH is connected to diencephalic structures. Some researchers have called for including diencephalic structures as key components of the hippocampal circuitry, playing crucial roles in its memory functions (Vann and Nelson 2015). This view is in line with our suggestion that even in mammals, the hippocampus plays an important role in some conscious processes (cetaceans may be an informative exception; Jacobs 2022). Interestingly, thalamo-hippocampal connections are reminiscent of the intricacy of thalamocortical connections, so considering the thalamo-hippocampal system in future research may shed light on conscious processes in all vertebrates (see Merker (2007) for subcortical involvement in human consciousness and Eichenbaum (2017) for the importance of the hippocampus).

We believe that the findings synthesized in this paper strengthen the suggestion that all vertebrates should be considered conscious beings since they all share GNW/UAL supporting structures (Allen 2013). According to this proposal, consciousness first emerged in the vertebrate lineage during the Cambrian (Ginsburg and Jablonka 2007b, 2010, 2019; Feinberg and Mallatt 2013, 2016), and selection for increasingly open-ended learning was the driving force leading to the evolution of the GNW/UAL architecture. Furthermore, we think that developing a separate and dedicated integrating system for long-term event memory was the first step that led mammals and birds on the path to imaginative experiencing. We therefore expect to see the effects of the co-evolution of new and better cognitive abilities, leading to new kinds of subjective experiencing in birds and mammals, to be reflected in the differentiation and the connectivity patterns of the hippocampus (work in progress).

### Predictions and future directions

#### The unity of consciousness

The presence of two separate sensory processing centers (for olfaction in the telencephalon and all other modalities in the midbrain), which independently affect perception, suggests that there could be a dissociation between UAL behaviors related to the different sensory modalities. For example, we expect that olfactory discrimination may remain intact even when discrimination involving other senses is obliterated. Hence, the prediction of Birch et al. (2020) regarding the co-development and co-evolution of integrated sensory information may have to be qualified, especially with regard to anamniotes. This, as we noted earlier, depends on the degree of sensory integration occurring in the tectum and pallium.

The unity of consciousness could also be studied by examining UAL tasks related to different sensory modalities in fish, by following changes in brain activity during and after anesthesia, and by investigating the effects of brain lesions. Lesioning dedicated sensory processing areas can lead to deficits in conscious perception of a single modality in all animals (such as blindsight in humans and other mammals). Here, we emphasize the predicted consequences of lesioning either the tectum or the telencephalon in basal fish, which have extensive (non-olfactory) sensory integration in their tectum and minimal (non-olfactory) sensory integration in their telencephalon before reaching the HH.

#### Testing ignition and feedback dynamics in anamniotes

Ignition dynamics have been proposed as a central marker (and mechanism) for differentiating conscious from non-conscious processes in humans (Mashour et al. 2020). For instance, non-conscious visual processing is characterized by early activity in the visual cortex, which does not propagate to frontal brain regions but quickly fades. In contrast, when visual information is perceived consciously, strong signals of later activity (∼200–300 ms) can be detected in parietal and frontal areas. This process is non-linear (there is either fast suppression or full ignition) and ignition activity is sustained, enabling feedback from frontal areas back to the visual cortex. Across many experiments, these ignition dynamics were consistently correlated with participants’ reports of conscious perception, and they are eliminated under conditions of inattention, distraction, and dreamless sleep.

This aspect of the GNW theory was recently tested in an adversarial collaboration, in comparison to Integrated Information Theory (IIT) (Cogitate Consortium et al. 2023; see also Vishne et al. 2022). Proponents of the two theories provided specific predictions for experimental outcomes that would reflect the underlying architecture proposed by each theory. Among many other interesting findings, it seems that ignition was identified during stimulus onset, but not stimulus offset. More broadly, there is an ongoing debate on the extent of processing necessary for a stimulus to be perceived consciously, with GNW theory requiring global involvement, while proponents of IIT and other theories considering localized processing to be sufficient. The ignition pattern (especially in the prefrontal cortex) predicted by Dehaene in Cogitate Consortium et al. 2023 was meant to reflect the global nature of conscious processing in the brain. The UAL theory is aligned with the GNW on this point, as it requires the integration of different functional networks into an activity pattern that includes evaluated sensory and prospective action mappings. However, the UAL theory is not committed to a specific pattern of global activity or ignition timing. Moreover, we would expect that if ignition-like activity could be identified in a wide variety of non-human animals, it would vary according to the task and the specific neuroanatomy of each species.

To our knowledge, no attempts have been made to identify ignition dynamics in anamniotes (though recent studies show that trace conditioning but not delay conditioning is disrupted by distractions in *Drosophila*; Grover et al. 2022). We expect to find such dynamics in all vertebrates. We think it would be especially interesting to record neural activity simultaneously in the fish HH and midbrain tectum, identifying instances in which tectal sensory activity is propagated to the HH in comparison to instances where it is not. To conduct such experiments, the experimental paradigm should allow for propagation variability. In human experiments, stimuli are presented around the threshold of conscious perception so that some trials are reported as perceived, and others as not perceived. This is achieved by very fast stimulus presentation, presentation in low-contrast conditions, masking (the stimulus is quickly preceded or followed by another one), and similar distracting interventions. Additionally, there must be an experimental option for report. Human subjects can answer verbally whether they experienced a stimulus, and this may be uncoupled from unconscious effects on behavior. For instance, a human subject may report that they have not seen a word flash on the screen but may still select the correct word when forced to make a choice. Report paradigms present a significant challenge to studying consciousness in non-human animals, but some progress has been made in primates and birds (Edelman and Seth 2009; Nieder et al. 2020; Crump and Birch 2022), and similar experimental paradigms can be used to investigate the effects of ignition in fish.

Recurrent connections enabling feedback activity are central to conscious experience and for maintaining percepts over time. In several primate experiments, researchers found that when monkeys were able to perceive a stimulus, this was predicted by frontoparietal feedback enhancing primary sensory area activity (Mashour et al. 2020, p. 782). In other words, success in a complicated perception task depended on activity in both the primary sensory area and activity originating from the frontal areas. Testing whether some (non-olfactory) perceptual tasks in anamniotes demand an intact HH, and whether this is due primarily to HH itself or to its enhancing effects (via recurrent connections) on the tectum, may elucidate the re-entrant dynamics involved.

Selectively targeting directional connections from one region to another may add more nuance to traditional lesion studies. Instead of ablating a whole pallial area (such as the HH), we suggest selectively severing or inactivating either the feed-forward connections from the midbrain tectum to the HH or the feedback connections leading from the HH to the tectum, all while maintaining the connections of both areas to motor subnetworks. According to the minimal GNW/UAL model suggested here, we expect that complex types of learning and perceptual discrimination will be disturbed or eliminated, while more limited learning will be maintained.

#### Testing UAL abilities in anamniotes

According to the UAL theory, all vertebrates, including the basal fish lineages, should display complex forms of associative learning, including trace conditioning, second-order conditioning, re-coding of stimulus value, and learning to discriminate among new and compound stimuli. Unfortunately, for some lineages (such as basal cartilaginous fishes) our current knowledge of complex learning is very limited, so assessing UAL in these lineages is needed to test this prediction. Since Ginsburg and Jablonka (2019) predict that limited (elemental) learning can occur subliminally and will not be significantly disturbed by distractors, whereas UAL will be abolished or significantly reduced, using distractors during UAL and limited learning in fish can directly test the link between UAL and consciousness. Such research could be further expanded to test species-specific abilities in the different sensory modalities and for the effects of deficits caused by selective lesions of GNW connections. Given that olfactory information reaches the HH without undergoing the extra stages of multimodal integration processing in the tectum and the preglomerular complex that are undergone by inputs from other modalities, the encoding of olfactory cues may be different than that of other sensory inputs. It will be interesting to investigate UAL learning under different conditions (with and without distractors) in the olfactory modality and compare it to UAL learning in other modalities.

#### Use of anesthesia in anamniotes

Anesthesia may be a fruitful avenue for testing different models of consciousness (Mashour and Alkire 2013; Mashour et al. 2020, p. 785). Almost all vertebrates display sleep–wake cycles and are influenced by anesthetic drugs (Wayson et al. 1976; Neiffer and Stamper 2009; Miyazaki et al. 2017). The GNW theory predicts that the effects of anesthesia that lead to loss of consciousness are mostly due to connectivity disruption between GNW hubs, while local processes may be spared. Mashour et al. (2020) point to studies showing prefrontal and parietal areas disrupted in anesthetic conditions, with a reduction in feedback activity and a complete loss of ignition dynamics. While many anesthetic studies have been conducted in mammals, much less is known about other vertebrate groups, especially anamniotes. It would be useful to record neural activity while animals undergo anesthesia, with particular emphasis on the HH and its relationship with other species-specific processing hubs. We predict similar disruption in the fish HH to that seen in the mammalian prefrontal and parietal cortex and loss of synchronization and feedback dynamics with other pallial and subpallial areas.

#### Going beyond anamniotes

This paper is limited to the discussion of jawed fish consciousness although, as Suzuki (2021) has noted, jawless fish have homologs of all the areas we specified and therefore can be considered minimally conscious. Although the homology of brain structures between vertebrates and invertebrates is not clear, functional and connectivity similarities between mammals and insects have been identified (Strausfeld and Hirth 2013), and UAL is found in some arthropods and the coleoid cephalopods (Ginsburg and Jablonka 2019). These invertebrates can be considered minimally conscious, and we expect them to have evolved a GNW-like functional architecture. Many of the experiments suggested here, such as the use of neural recording techniques coupled with anesthetics, selective inhibition of feedback or feed-forward connections, and behavioral paradigms of open-ended associative learning, report, and masking, can be applied to invertebrates. These will enable researchers to estimate the kinds of subjective experiences available to different lineages and the scope and richness of consciousness in the animal kingdom.

Once minimal consciousness evolved and vertebrates diverged and diversified, new modes of consciousness, such as imaginative consciousness, emerged. Mammals and birds underwent several dramatic ecological shifts and developed larger brains with new and improved capacities and highly specialized structures. We think that in mammals, the shift of sensory processing areas from the midbrain tectum to the growing neocortex that co-evolved with the specialization of hippocampal and cortical connectivity patterns is a crucial step in the development of mammalian cognition and consciousness. The evolution of birds followed a different route, but there is a striking convergence in birds’ and mammals’ behavior and cognitive abilities, including, in some lineages, episodic-like memory and some imaginative planning (Clayton 2017; Pika et al. 2020). Researchers have also identified convergent neural mechanisms, including those predicted by the GNW theory (such as extensive recurrent feedback connections and long-range connections within and outside the telencephalon; Puelles et al. 2001; Jarvis et al. 2005; Niu et al. 2022). The framework developed in this paper can be expanded to studying these shifts and tracing the processes that led to imaginative consciousness and finally to the symbol-based consciousness of humans.

In this paper, we have discussed the evolutionary origin of the GNW architecture, which, we suggested, is an essential part of the dynamic architecture that underlies minimal consciousness in vertebrates. We believe that a similar evolutionary analysis may be applied to other leading theories of consciousness that are centered on humans, such as IIT (Tononi et al. 2016) and recurrent processing (Lamme 2018). Since an origin-focused evolutionary approach can uncover fundamental principles of minimal consciousness without being misled by human-specific elaborations and specializations, we expect that such analyses will both suggest better differentiation among different theories and highlight their common ground.
